# Photosynthetic Control of Arabidopsis Leaf Cytoplasmic Translation Initiation by Protein Phosphorylation

**DOI:** 10.1371/journal.pone.0070692

**Published:** 2013-07-24

**Authors:** Edouard Boex-Fontvieille, Marlène Daventure, Mathieu Jossier, Michel Zivy, Michael Hodges, Guillaume Tcherkez

**Affiliations:** 1 Institut de Biologie des Plantes, CNRS UMR 8618, Saclay Plant Sciences, Université Paris-Sud, Orsay, France; 2 Plateforme PAPPSO, UMR de Génétique Végétale, Ferme du Moulon, Gif sur Yvette, France; 3 Institut Universitaire de France, Paris, France; The John Curtin School of Medical Research, Australia

## Abstract

Photosynthetic CO_2_ assimilation is the carbon source for plant anabolism, including amino acid production and protein synthesis. The biosynthesis of leaf proteins is known for decades to correlate with photosynthetic activity but the mechanisms controlling this effect are not documented. The cornerstone of the regulation of protein synthesis is believed to be translation initiation, which involves multiple phosphorylation events in Eukaryotes. We took advantage of phosphoproteomic methods applied to *Arabidopsis thaliana* rosettes harvested under controlled photosynthetic gas-exchange conditions to characterize the phosphorylation pattern of ribosomal proteins (RPs) and eukaryotic initiation factors (eIFs). The analyses detected 14 and 11 new RP and eIF phosphorylation sites, respectively, revealed significant CO_2_-dependent and/or light/dark phosphorylation patterns and showed concerted changes in 13 eIF phosphorylation sites and 9 ribosomal phosphorylation sites. In addition to the well-recognized role of the ribosomal small subunit protein RPS6, our data indicate the involvement of eIF3, eIF4A, eIF4B, eIF4G and eIF5 phosphorylation in controlling translation initiation when photosynthesis varies. The response of protein biosynthesis to the photosynthetic input thus appears to be the result of a complex regulation network involving both stimulating (e.g. RPS6, eIF4B phosphorylation) and inhibiting (e.g. eIF4G phosphorylation) molecular events.

## Introduction

Intense efforts are currently devoted to disentangle the regulation of protein biosynthesis in plant organs with the aim to increase the protein fraction or the nitrogen content in crops. In addition to nitrogen nutrition, metabolic commitments and transcriptional control, mRNA translation is believed to be of importance to regulate protein synthesis and protein content of plant tissues. Plants are phototrophic organisms and thus their translational activity is strongly influenced by light and photosynthesis. Pioneering studies with isotopic labeling showed that in mature leaves, gross protein synthesis (incorporation of ^15^N in proteins) was larger in the light than in the dark [1] and in *Chlorella pyrenoidosa* (single celled green alga), steady-state photosynthesis has been found to be associated with ^14^C-labeling in proteinaceous amino acids [2]. More recently, molecular studies have shown that in plant leaves, light stimulates translational activity of photosynthesis-related mRNA (ferredoxin) [3] and furthermore, a larger fraction of polysomal ribosomes has been found in the light compared to the dark [4]. In leaves, isotopic labelling (^14^CO_2_) has demonstrated that an increasing proportion (from 10 to 24% of net fixed carbon) of carbon is allocated to protein synthesis as photosynthesis increases from low to high [CO_2_] conditions, demonstrating a positive effect of photosynthetic input (CO_2_ mole fraction) on gross protein synthesis [5]. Nevertheless, little is known about the mechanisms by which photosynthesis influences translation and overall protein production.

Quite generally, protein phosphorylation appears to play a crucial role in the regulation of translational initiation. A key-step of translational control seems to be the phosphorylation of the ribosomal protein S6 (RPS6), since alterations of its phosphorylation level have drastic effects on growth and polysome formation [6], [7]. Recent proteomic characterization of ribosomal phosphorylation patterns showed a significant increase of the phosphorylation level of RPS6 as well as two other ribosomal proteins (RPP1 and RPL29-1) in the light when compared to the dark [8]. RPS6 is known to be phosphorylated by S6 kinase (S6K) which in turn may be phosphorylated by other kinases such as PDK1 and the TOR/RAPTOR complex [9], [10]. Translation initiation in the cytoplasm begins with the recognition of the cap structure (5′ end of mRNA) by the initiation factors eIF4E, eIF4G, eIF4B and the RNA helicase eIF4A. The 40S ribosomal subunit binds an eIF2-containing complex (eIF2, GTP and Met-tRNA) and the initiation factors eIF1, eIF1A, eIF3 and eIF5, forming the 43S initiation complex. The cap-binding complex interacts with the 43S initiation complex and allows mRNA scanning and the correct positioning at the start codon. eIF5B eventually promotes joining of the 60S ribosomal subunit. At each of these steps, eIF-phosphorylation is crucial for the control of initiation and there are multiple phosphorylation sites on these proteins, which can either stimulate or repress translation (for a review, see [11]). The phosphorylation of eIF1, eIF2β, eIF3c and eIF5 by CK2 favours the assembly of the complex that binds the 40S ribosomal subunit *in vitro* [12], [13]. Phosphorylation of eIF4B and poly-A binding protein (PABP) promotes their interaction and the formation of a circular mRNA structure that stimulates translation initiation [14]. In mammalian cells, the alteration of eIF4E phosphorylation compromises mRNA cap recognition, however if eIF4E phosphorylation occurs in plants the equivalent phosphorylated Ser residue in the primary sequence is missing [15]. Similarly, eIF4G and eIF(iso)4G, which participate to mRNA binding and interact with eIF4B, might be controlled by phosphorylation but this is at present uncertain due to the absence of the phosphorylation motif for binding the MNKI kinase [16]. Phosphorylation of eIF2α by GCN2 seems to down-regulate translation by disfavouring the eIF2B-catalyzed exchange of GDP for GTP [16], [17]. The current knowledge of this complex orchestration mostly stems from in vitro studies, characterization of mutants or use of stressful conditions (e.g., anoxia). But quite critically, the way by which eIF phosphorylation is influenced by natural light/dark conditions and photosynthetic conditions is not well documented.

As an aid in clarifying the nature and the phosphorylation of molecular actors involved in photosynthesis-driven translational control, we have investigated the phosphorylation of ribosomal proteins and initiation factors in *Arabidopsis* leaves in different photosynthetic contexts: various CO_2_ conditions (ordinary [CO_2_] (380 ppm), high [CO_2_] (1000 ppm), low [CO_2_] (100 ppm)) in the light and ordinary CO_2_ in the dark. We took advantage of a gas-exchange system with liquid N_2_ spraying for instant sampling and nanoLC-MS/MS based phosphoproteomics to characterize phosphorylated peptides in rosette leaves. We report 11 new phosphorylation sites in eIF proteins and 14 new sites in ribosomal proteins (RPs), describe significant CO_2_-dependent protein phosphorylation patterns and show concerted changes in 13 eIF phosphorylation sites and 9 ribosomal phosphorylation sites. Our results suggest a key role of eIF and RP phosphorylation in photosynthesis-driven regulation of mRNA translation in leaves.

## Results

### Photosynthetic Conditions and Sampling

**Figure 1:**
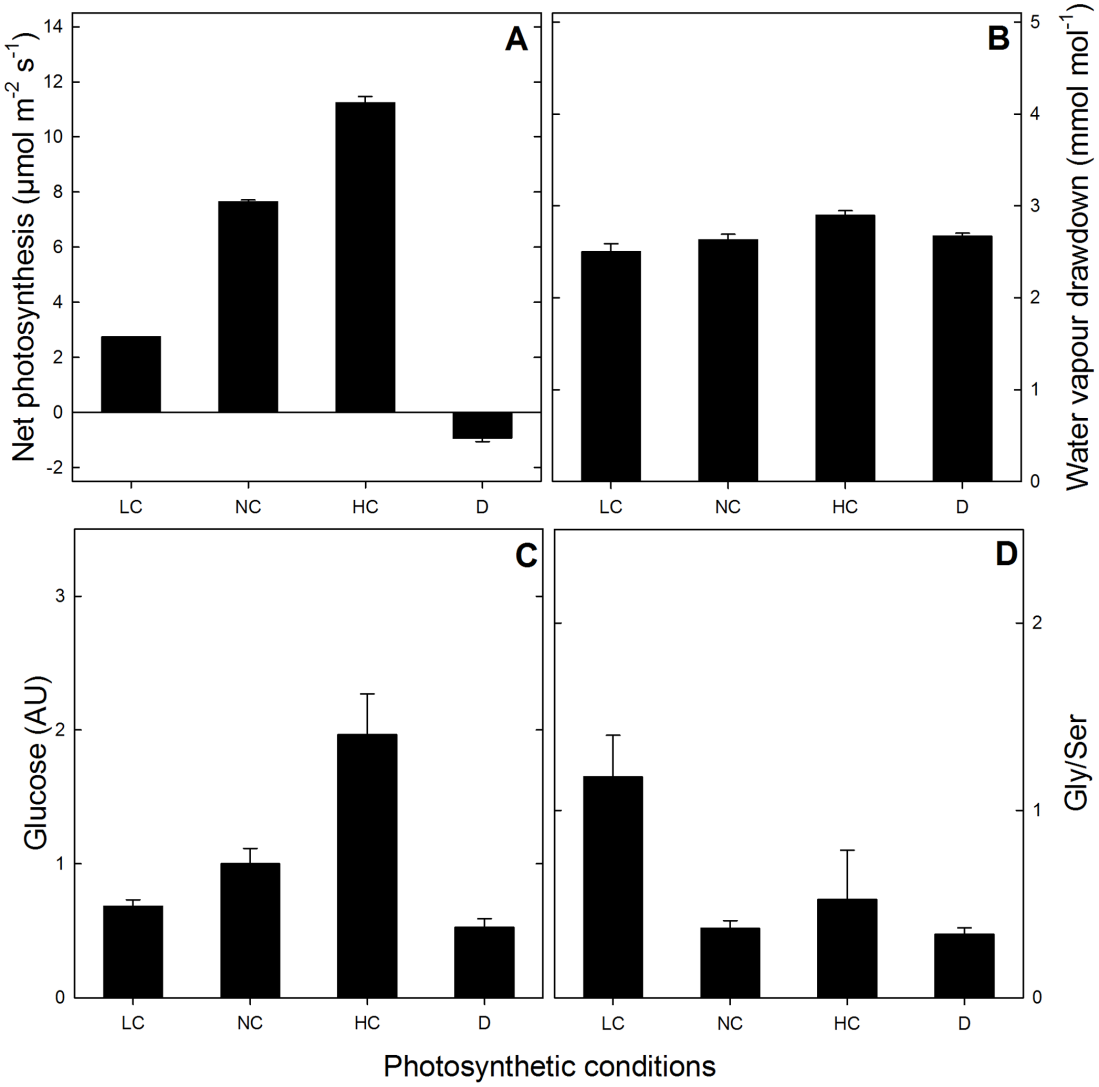
Photosynthetic parameters. leaf net photosynthesis (**A**), leaf-to-air water vapour drawdown (**B**), leaf glucose content (**C**) and Gly-to-Ser ratio (**D**), under the different photosynthetic contexts used (LC, NC and HC: 100, 380 and 1000 µmol mol^−1^ CO_2_; D: darkness).

Short-day grown *Arabidopsis* rosettes were placed in a purpose-built chamber connected to a gas-exchange system, as described in the *Material and Methods*. CO_2_ and water vapour (H_2_O) were monitored so as to calculate photosynthesis and transpiration. After four hours at a photosynthetic steady-state at the desired CO_2_ mole fraction, leaf rosettes were sprayed with liquid nitrogen and sampled. Sampling in the dark was carried out on dark-adapted leaves, that is, after two hours in darkness following 4 hours of steady photosynthesis at ordinary CO_2_ (380 ppm). Protein composition and phosphorylation were analysed by nanoLC-MS/MS. Figures 1A and 1B show steady net photosynthesis rates and leaf-to-air water vapour draw-down levels, respectively. There was a very clear effect of CO_2_ on photosynthesis demonstrating that the four types of samples analysed here (ordinary, low and high CO_2_ and darkness) corresponded to strongly different photosynthetic contexts. This CO_2_ effect was independent from water deficit since the water vapour draw-down between evaporation sites and atmosphere remained constant (Figure 1B). Photosynthetic metabolism (photosynthate production) was reflected by the glucose content which correlated with the photosynthesis rate (Figure 1C). The quotient of the two key photorespiratory metabolites Gly and Ser, which correlates with O_2_ fixation (photorespiration activity), was indeed higher at low CO_2_ (Figure 1D, LC).

### LC-MS Analysis of Leaf Proteins

For proteomic characterization, protein samples were digested by trypsin and then analysed as described in [18]. Peptides were methylated via formylation with labeled (deuterated) or non-labeled formaldehyde and reduction with cyanoborohydride. Non-labeled and labeled peptides were mixed (the condition of interest (non-labeled) and the mix of all samples as a reference (labelled) mixed with a 1∶1 mixing ratio, see *Material and methods*), and underwent a SCX (Strong Cation Exchange) chromatography. Collected fractions were enriched in phospho-peptides by IMAC (Immobilized ion Metal Affinity Chromatography) and then analysed by nanoLC-MS/MS. The quantity of proteins was determined by direct analysis (no SCX and labeling). (Phospho)peptides were identified with X!Tandem [19] and quantified with MassChroQ [20]. Table 1 summarizes the number of phosphopeptides identified using nanoLC-MS/MS, with the complete list of phosphorylated peptide sequences in Table S1. 156 ribosomal proteins were detected, among which 33 phosphorylated ribosomal proteins were found, represented by 45 phosphopeptides (for a complete description of RP and eIF proteins identified, see Table S2). 25 eIFs were detected, with 15 phosphorylated proteins represented by 28 phosphopeptides. The statistical analysis of peptide quantity in samples (three independent biological replicates were analysed for each experimental condition) indicated no difference between the CO_2_ and dark treatments. Therefore, the changes in phosphopeptide abundance described below are strictly related to changes in the phosphorylation ratio.

**Table 1:** Eukaryotic ribosomal protein and initiation factor phosphopeptides identified by nanoLC-MS/MS.

Protein	Gene locus	Phosphopeptidesidentified^a^	Phosphorylationsites	New sites^b^	Significantsites^c^	Insignificantsites	Sites punctuallydetected^d^
**RPS2C**	At2g41840	3	2	1	0	Ser 258, Ser 273 or Thr 275or Ser 276	0
**RPS3aA**	At3g04840	2	2	0	0	Ser 69, Ser 236	0
**RPS3aA, RPS3aB**	At3g04840, At4g34670	1	1	1	0	0	Ser177
**RPS6A**	At4g31700	7	4	0	Ser 237, Ser 240, Ser 247	0	Ser 249
**RPS6B**	At5g10360	4	2	0	Ser 237, Ser 240	0	0
**RPS6A, RPS6B**	At4g31700, At5g10360	3	2	1	Ser 229, Ser 231	0	0
**RPS9B**	At5g15200	1	1	0	0	0	Ser 68
**RPS10A**	At4g25740	1	1	1	0	0	Ser116
**RPS14A**	At2g36160	1	1	1	Ser 19	0	0
**RPS14B**	At3g11510	1	1	0	0	Ser 19	0
**RPS17**	At1g79850	1	1	0	0	0	Thr 115 or Ser 117
**RPS27A, RPS27B, RPS27C**	At2g45710, At3G61110, At5g47930	1	1	0	0	Ser 29	0
**RPP0B**	At3g09200	1	1	0	0	Ser 305	0
**RPP0C**	At3g11250	1	1	0	0	Ser 308	0
**RPP1A, RPP1C**	At1g01100, At5g47700	1	1	1	0	0	Ser 10
**RPP1A, RPP1B, RPP1C**	At1g01100, At4g00810, At5g47700	2	1	0	0	Ser 102	0
**RPP2A**	At2g27720	1	1	1	0	Ser 95	0
**RPP2B**	At2g27710	1	1	1	0	Ser 80	0
**RPP2A, RPP2B, RPP2D**	At2g27720, At2g27710, At3g44590	2	1	0	0	Ser 120	0
**RPP3A, RPP3B**	At4g25890, At5g57290	2	1	0	0	0	Ser 90
**RPL3A**	At1g43170	3	3	3	0	Ser 28	Ser 139, Ser 377
**RPL6B, RPL6C**	At1g74060, At1g74050	1	1	1	0	Ser 52	0
**RPL11A, RPL11B, RPL11C, RPL11D**	At2g42740, At3g58700, At4g18730, At5g45775	1	1	0	0	Thr 46	0
**RPL13D**	At5g23900	1	1	0	Thr 138	0	0
**50S ribosomal protein L1**	At363490	1	2	1	0	0	Ser32, Ser 35
**RACK1B**	At1g48630	1	1	1	0	0	Ser 285
**eIF5**	At1g77840	1	1	0	0	0	Ser 201
**eIF5A2**	At1g26630	2	1	0	Ser 2	0	0
**eIF5A3**	At1g69410	2	1	1	Ser 2	0	0
**eIF4A1**	At3g13920	1	1	0	Ser 4	0	0
**eIF4A1, eIFA2**	At3g13920, At1g13020	1	1	0	Thr 145	0	0
**eIF4A3**	At1g72730	1	1	0	Thr 147	0	0
**eIF4B1**	At3g26400	3	3	1	Ser 480	Ser 462	Thr 239
**eIF4B2**	At1g13020	5	5	3	Ser 475, Ser 489	Thr 283, Ser 493	Ser 136
**eIF4G**	At3g60240	7	7	3	Thr 177 or Ser 178, Ser 178	0	Ser 530, Ser 710, Ser 1353, Ser 1508, Ser 1527
**eIF3B1, eIF3B2**	At5g27640, At5g25780	2	2	1	Ser 684	Ser 273	0
**eIF3 subunit 7**	At4g20980	1	1	0	Thr 74	0	0
**eIF3 subunit 7**	At5g44320	1	1	1	Thr 70	0	0
**eIF3c1**	At3g56150	1	1	1	Ser 40	0	0
**eIF2B beta**	At3g07300	1	1	1	0	Ser 173	0
**eIF2B delta**	At5g38640	10	9	3	Ser 127	Ser 108, Ser 220	Ser 88, Ser 91, Ser 93, Ser 126 or Ser 127, Ser 141 or Ser142 or Ser 143, Ser 218 or Thr 219 or Ser 220
**TruB**	At5g14460	1	1	0	Ser 132	0	0
**TOTAL**		85	73	29	24	23	26

a- Phosphopeptides sequences are listed in Table S2.

b- A phosphorylation site is considered as new when absent from refs. [8] and [11] and PhosPhAt 4.0 database(http://phosphat.mpimp-golm.mpg.de/).

c- Phosphorylation patterns are presented in Figures 3 and 4.

d- By ‘punctually’, we mean occasional detection of phosphorylation, with no clear pattern.

### Phosphopeptides and Phosphorylation Sites

**Figure 2:**
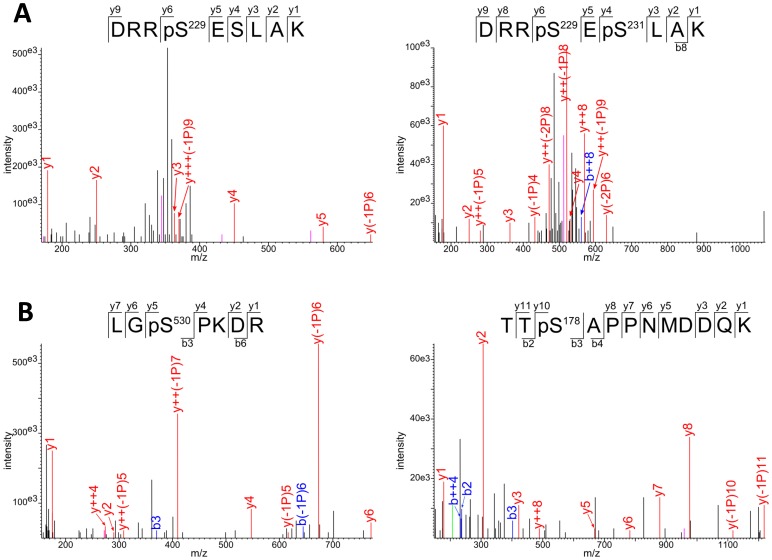
Identification of Ser 229 and Ser 231 in RPS6A/B (A) and Ser 178 and Ser 530 in eIF4G (B) by mass spectrometric sequencing of two phosphorylated peptides. Spectra of methylated and phosphorylated peptides show b (N-terminal) and y (C-terminal) fragment-ions as displayed in the sequence (top of each spectrum). Lower case p indicates the phosphate group. Phosphorylation is localized according to the pattern of the fragment-ions containing phosphate and fragment-ions with phosphate loss. Ions showing a neutral loss of H_3_PO_4_ and 2×H_3_PO_4_ are labelled with “-1P” and “-2P” respectively. Fragment from neutral losses are coloured in pink, and fragment ions are coloured in green. Parental ion fragments are, as shown in insets: DRRpSEpSLAK (*m/z* 643.31177, *z* = 2), DRRpSESLAK (*m/z* 402.55502, *z* = 3), LGpSPKDR (*m/z* 458.75925, *z* = 2) and TTpSAPPNMDDQK (*m/z* 724.83307, *z* = 2). They were identified in 6, 20, 1 and 76 spectra, respectively.

Phosphopeptides identified in RPs and eIFs are listed in Table 1, in which significant (i.e., with statistically significant changes under photosynthetic/light conditions), insignificant and punctual (i.e., punctually phosphorylated with little repeatability) sites are distinguished. Phosphorylation sites were mapped using MS spectra and searching with the MASCOT engine, thus giving obvious phosphorylated residues in peptides. In some instances, however, ambiguous cases occurred, in which the nature of the phosphorylated residue could not be determined (two undistinguishable possibilities). This was the case for peptides from RPS6A/RPS6B, RPS2C, RPS17 and eIF4G that include two phosphorylatable Ser residues or both Ser and Thr residues (Table S2). New phosphorylation sites (absent from PhosPhAt 4.0 (http://phosphat.mpimp-golm.mpg.de/) and from [21] and [8]) were detected in at least 16 ribosomal proteins, including in the ribosomal proteins RPS6A and/or RPS6B at Ser 229 (with mono- and bis-phosphorylated peptides DRRpS^229^ESLAK and DRRpS^229^EpS^231^LAK, respectively, Figure 2). In this case, the specific nature of the ribosomal protein RPS6A or B could not be solved due to their close sequence identity. We also detected a new phosphopeptide located in the activation loop of the S6 kinases S6K1 and S6K2 (SNpS^290^MCGTTEYMAPEIVR). Other new sites located in eIFs are listed in Table 1 and amongst them, the most significant are Ser 178 (or Thr 177) and Ser 530 in eIF4G (Figure 2). We did not detect any phosphorylated sites in eIF4E and PABP (Poly-A Binding Proteins). Amongst the components of the eIF2B complex (a guanidine nucleotide exchange factor which catalyzes the substitution of hydrolysed GDP for GTP in eIF2), no phosphopeptide was detected for eIF2Bα, eIF2Bγ and eIF2Bε while eIF2Bβ was phosphorylated at Ser 173 (SADKSpS^173^LTR) and eIF2Bδ in 10 different Ser residues (Table 1).

### Phosphorylation Patterns

**Figure 3:**
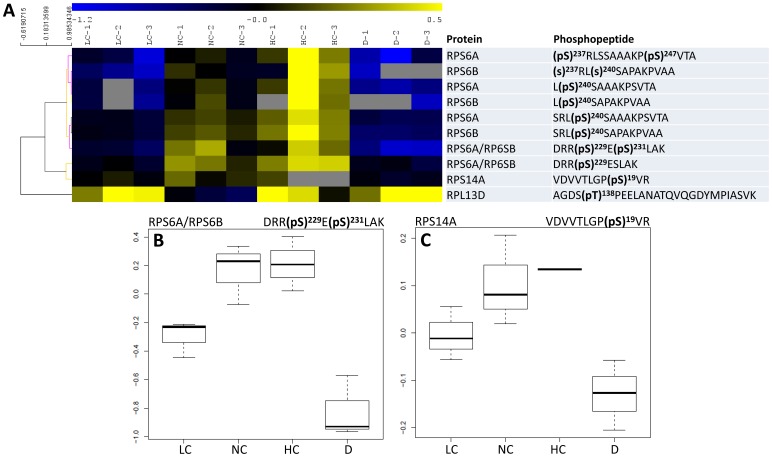
Phosphorylation pattern of ribosomal proteins. **A**, Heat map representation of the phosphorylation level of significant phosphopeptides (phosphorylated peptides that showed statistically significant changes with conditions). A hierarchical clustering analysis is shown on the left. All significant phosphopeptides had a very similar pattern, except for RPL13D, which was minimally phosphorylated under ordinary conditions (NC). Unavailable data (non-detected peptides) are indicated with a grey cell. **B** and **C**, Detailed phosphorylation pattern of RPS6A/B and RPS14A. LC, NC, HC and D: low, normal and high CO_2_ and darkness.

**Figure 4:**
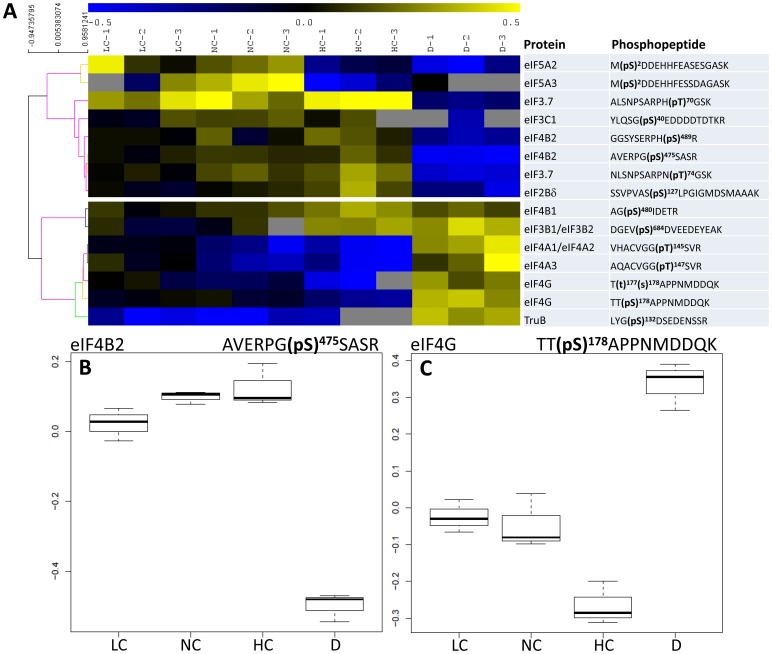
Phosphorylation pattern of initiation factors eIFs. **A**, Heat map representation of the phosphorylation level of significant phosphopeptides. A hierarchical clustering analysis is shown on the left so as to separate photosynthesis or light-stimulated (top) and -inhibited (bottom) phosphorylation events. Unavailable data (non-detected peptides) are indicated with a grey cell. **B** and **C**, Detailed phosphorylation pattern of eIF4B2 and eIF4G. LC, NC, HC and D: low, normal and high CO_2_ and darkness.

Nine phosphorylation sites in ribosomal proteins and 13 phosphorylation sites in eIFs were significantly affected (*P*<0.04) by conditions: light/dark and/or photosynthetic activity (CO_2_ mole fraction). Their phosphorylation patterns are shown in Figures 3 and 4. Several phosphorylation sites in ribosomal proteins were affected by both light/dark and photosynthesis conditions, such as Ser 229 and 231 in RPS6A and/or RPS6B, Ser 19 in RPS14A and Thr 138 in RPL13D (Figure 3A and 3B). In the latter case, the photosynthetic effect was not monotonous and light decreased Thr 138 phosphorylation (at 380 ppm CO_2_). Most sites in eIFs were significantly affected by light/dark conditions, except for eIF4B1 at Ser 480; by contrast, this site was significantly affected by CO_2_ mole fraction. Most sites were positively influenced by CO_2_ mole fraction (i.e. there was a positive correlation between phosphorylation and photosynthetic activity), except for eIF4G, eIF4A and eIF5A (Figure 4A). That is, the components of eIF4F were either not phosphorylated in our conditions (eIF4E) or responded negatively to photosynthetic conditions (eIF4G, eIF4A). 17 and 5 sites in ribosomal proteins and eIFs, respectively, were constantly phosphorylated, with no significant change with light/dark or CO_2_ mole fraction. For example, this was the case of Ser 52 in RPL6C and Ser 4 in eIF4A1 (Table S1). The pseudo-uridylate synthase (TruB, enzyme which post-transcriptionnally isomerizes uridine residues in t-RNA) was affected by light/dark conditions (more phosphorylated at night at Ser 132) but not by photosynthesis, while eIF2Bδ at Ser 127 was contrarily affected (more phosphorylated in the light, significant photosynthetic effect) and eIF2Bβ (Ser 173) was not significantly affected by any condition. That is, the phosphorylation pattern of the eIF2B complex did not appear to be simple, with contrasted effects on individual subunits.

## Discussion

The phosphorylation status of eIFs and RPs is known to be crucial in regulating protein interactions and activity for translation initiation [11]. Here, we show multiple phosphorylation sites and protein phosphorylation patterns affected by light and photosynthetic (CO_2_) conditions.

### RP Phosphorylation

In eukaryotic cellular systems examined so far (mammals, yeast and plants), cytosolic RPs have been found to be phosphorylated, allowing a fine control of translation. RPS6 activity seems to be controlled by phosphorylation (for a review, see [22]). In mammalian cells, the TOR kinase phosphorylates the S6K kinase that in turn phosphorylates RPS6. Homologs of TOR and S6K exist in plants and RPS6 has indeed been found to be phosphorylated [23], [24]. A recent investigation of RP phosphorylation in *Arabidopsis* leaves in the light and in the dark has further shown that RPP1A/B/C, RPS6A/B, RPP0B, RPS2C and RPL29A are phosphorylated and RPP1A/B/C, RPS6A/B, and RPL29A respond to light/dark conditions: RPS6A/B appeared to be significantly more phosphorylated in the light while RPP1A/B/C and RPL29A appeared to be slightly but insignificantly more phosphorylated in the light [8]. Here, we show that in addition to RPP1A/B/C, RPS2C, RPP0C and RPS6A/B, 25 other ribosomal proteins can be phosphorylated (Table 1), among which two are significantly affected by light (RPS14A and RPL13D). The phosphorylation of RPs of the small subunit (RPS6 and RPS14) further appeared to correlate with photosynthetic activity (Figure 3). Changes in phosphorylation of RPs caused by environmental or hormonal conditions have been found to occur in maize roots under hypoxia [23] and anticipated in plant lines with altered TOR or S6K kinases [9], [25]. During the light period and photosynthesis, which are associated with an increased translation activity (see *Introduction*), the stimulation of translational activity is thus likely to be associated with a pronounced phosphorylation of RPs, thereby promoting the formation of the initiation complex.

### eIF Phosphorylation

The phosphorylation of several eIFs is believed to be of considerable importance in triggering translation initiation [26], [27]. Multiple phosphorylations of several eIFs by CK2 are required for the formation of the mRNA-binding complex containing eIF4A, eIF4B, eIF4F and eIF5 [12]. To date, eIF4A has been found to be increasingly phosphorylated and eIF4B dephosphorylated in response to stressful environmental changes in plant cells [28], [29], [30]. eIF3c has been shown to be phosphorylated by CK2 [13] although no apparent effect of eIF3c phosphorylation on translation has been detected [31].

Here, we show that several eIF3 (eIF3a, eIF3b, eIF3c), eIF4 (eIF4A, eIF4B, eIF4G) and eIF5 (eIF5A2, eIF5A3) proteins are phosphorylated and many phosphorylation sites responded significantly to light/dark and photosynthesis conditions (Figure 4). Importantly, eIF4A and eIF4G were found to be less phosphorylated at high photosynthetic rates and more phosphorylated in the dark. It is plausible that eIF4A and eIF4G phosphorylation inhibits day-time translation or that the phosphorylation is not required under ordinary, non-stressful conditions (or alternatively, that photosynthate deprivation in the dark causes eIF4A and eIF4G phosphorylation). All phosphorylation sites of eIF4B respond positively to photosynthesis suggesting a reverse pattern (eIF4B phosphorylation required for ordinary translation). In our study, eIF4E, eIF(iso)4E and eIF(iso)4G did not appear to be phosphorylated, despite the fact that their involvement has proven important in selecting translated mRNAs [32]. eIF3c, eIF5A2 and eIF5A3 were found to be more phosphorylated in the light compared to the dark, with little photosynthetic effect. The interaction of eIF3c with eIF5 is believed to be crucial for AUG recognition along the mRNA and therefore, these eIFs (and eIF3c phosphorylation at Ser 40) are probably essential at all times when translation is active (daytime). We further show that other eIF3s are phosphorylated (eIF3B1/2), implying that eIF3 activity might also require phosphorylation at other sites. It should nevertheless be recognized that phosphorylation of eIF5A has been found to favour eIF5A sequestration in the nucleus and thus it has been hypothesized to repress translation [33], [34]. The individual effects of multiple phosphorylation events in eIFs on translation are thus presently difficult to appreciate but here, the clear effect on RPS6 and eIF4B phosphorylation undoubtedly reflects translation enhancement at increased photosynthetic rates.

### New Phosphorylation Sites

Most phosphorylation sites in RPs described here (Table S1) are novel, with the notable exception of Ser 231, Ser 237 and Ser 240 in RPS6A/B [8]. We found the new phosphorylation site Ser 229, which cannot be unambiguously attributed to RPS6A or RPS6B (Figure 2). eIF3c is associated with a phosphorylation site at Ser 40, which has been anticipated in *Arabidopsis* using sequence alignment with wheat (Ser 53) [12]. eIF4B is phosphorylated at Ser 422 in mammals but this site does not exist in *Arabidopsis* [35]. We found instead a phosphorylation site at Ser 462 in eIF4B1 and Ser 475 in eIF4B2 (and three other sites, Table 1). The three phosphorylation sites that vary significantly under our conditions (Ser 480 in eIF4B1, Ser 475 and Ser 489 in eIF4B2) seem to be conserved in higher plants (such as *A. lyrata, Populus trichocarpa, Glycine max* and *Vitis vinifera*). eIF4G appeared to be phosphorylated in the N-ter region as we found phosphorylation sites at Thr 177 and/or Ser 178. However, this region is not associated with a clear function in translation [29], [36]. We further found two new phosphorylation sites (Ser 530 and 1353) which might influence eIF4G activity (these Ser residues might be in regions interacting with eIF3 and eIF4E as in yeast [37]). eIF5A3 has been shown to be phosphorylated at Ser 2 in maize [33], [34] and the same site is found here (Table 1).

### Translational Control and Photosynthesis

**Figure 5:**
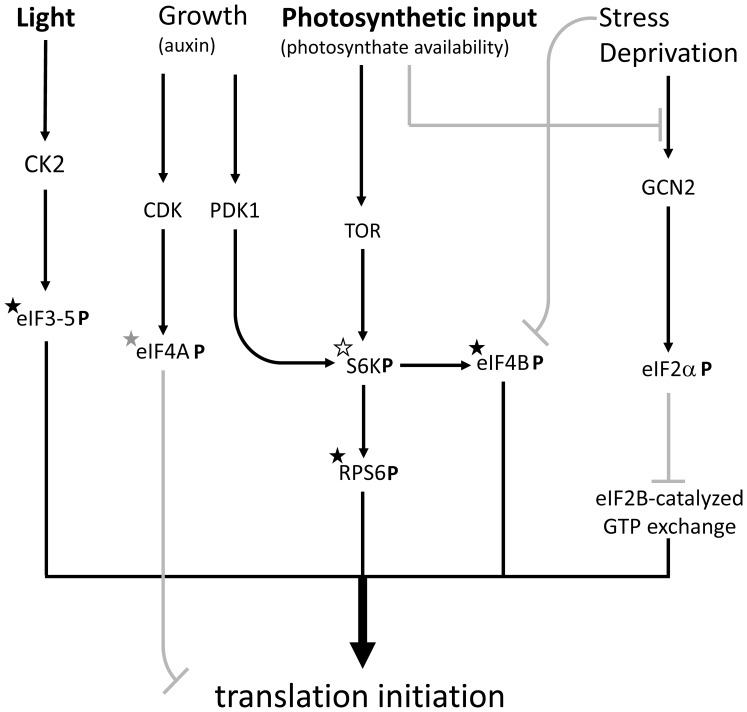
Tentative summary of protein phosphorylation events involved in translation initiation during photosynthesis, with activating (black) and repressing (grey) effects. Phosphorylated proteins are indicated with the symbol **P**. Those associated with phosphopeptides detected in the present study are indicated with a star, with the phosphorylation level that either correlates (black star), anti-correlates (grey star) or stays constant (white star) with photosynthesis. This figure is simplified and does not mention all molecular actors (such as eIF4G and eIF2Bδ).

Considering the whole data set obtained here, clear phosphorylation patterns were observed in key actors of translation initiation (RPs, eIFs) likely reflecting stimulation of daytime translation (compared to the dark). There is a considerable literature showing that nitrogen metabolism and nitrate assimilation occur during daytime in leaves (reviewed in [38]) while dark respiration is (partly) fed by protein degradation and amino acid recycling [39]. That is, metabolic imperatives caused by light/dark alternation are so that in leaves, gross protein synthesis and translational activity is more important in the light. Our results suggest that translation initiation is stimulated via phosphorylation of RP proteins, eIF2Bδ, eIF3 and eIF4B and dephosphorylation of repressing sites in eIF4A and eIF4G when photosynthesis increases. The molecular mechanism and rationale of this regulation remain to be elucidated, though. We suggest that important cellular kinases are responsible for this pattern (Figure 5). In fact, eIF2, eIF3c, eIF4B and eIF5 are phosphorylated by CK2 which in turn seems to be activated by light phase duration [40] and photomorphogenesis [41] and thus presumably, might be activated during active photosynthesis. In mammals, eIF4B is phosphorylated by the S6K kinase (which also phosphorylates RPS6) and ORF45 of the herpes virus, and this stimulates translational activity [42]. Despite the fact that the phosphorylated site of mammalian eIF4B is not conserved in plants, eIF4B phosphorylation probably stimulates translation initiation. Furthermore, plants have two S6K isoforms (S6K1 and S6K2) that are activated by PDK1 which is in turn activated by auxin response and growth [43]. S6K is also activated by the TOR-RAPTOR signalling pathway and, perhaps, SnRK1 under specific metabolic conditions (photosynthate and sugar availability) [9], [44]. By contrast, GCN2-catalyzed phosphorylation of eIF2α (from which no phosphopeptide was detected here) is stimulated under deprived or stressful conditions [45]. Taken as a whole, there is probably a balance between the stimulation of translation initiation caused by favourable nutritional conditions (photosynthesis) and the repression caused by cell division (e.g., CDK-dependent phosphorylation of eIF4A, [46]) or stress (e.g., GCN2-catalyzed phosphorylation of eIF2α) (Fig. 5).

### Perspectives

Our understanding of translation initiation in plants is still rather incomplete and further work is needed to disentangle the specific role of individual eIFs and their associated phosphorylation. In that regard, our model in Figure 5 is very crude and probably not fully representative. Future high-throughput sequencing and proteomics are warranted to yield substantial pieces of information on translational control in response to environmental conditions. Our data suggest that natural nutritional conditions influence translation *in folio*. This is probably of prime importance *in situ* (in the field) since changes in CO_2_ mole fraction occur quite frequently due to, for example, stomatal closure or diurnal CO_2_ changes in the ecosystem atmosphere. Some uncertainty nevertheless remains as to the targets of such a translational regulation: since the use of different eIFs modifies mRNA affinity (e.g., eIF4E versus eIF(iso)4E), the nature of mRNA selected for increased translation during photosynthesis is probably finely adjusted. Future translatomics (and polysome) analyses or ^15^N-labeling followed by protein-specific isotopic analyses would be required to describe the full translational picture of photosynthetising leaves.

## Materials and Methods

### Plant Material and Gas-exchange

After sowing on potting mix, *Arabidopsis* (Col-0 ecotype) plantlets were transplanted to individual pots and grown in a controlled environment (growth chamber) under 8∶16 h light/dark (short days) at an irradiance of approximately 100 µmol m^−2^ s^−1^, 20/18°C day/night temperature, 65% humidity and nutrient solution (1 g L^−1^ PP14-12-32, [Plant-Prod, Puteaux, France] supplemented with 20 µL L^−1^ fertoligo L [Fertil, Boulogne-Billancourt, France]) twice a week. Gas-exchange were carried out with a purpose-built cuvette adapted to three *Arabidopsis* rosettes connected to the sample channel of the Li-Cor 6400 *xt* (Li-Cor, Lincoln, USA). Water vapour in inlet air was fixed (dew point temperature 11.6°C) with a dew-point generator Li-610 (Li-Cor, Lincoln, USA). Air temperature in the chamber was maintained with a water-bath. Leaf rosettes were separated from the below-ground part and soil of the pot by a plexiglass wall (with specific holes for collars) so as to avoid alteration of gas-exchange by soil and root respiration. The upper wall of the leaf cuvette was made of a tight polyvinyl chloride film allowing instant sampling by liquid N_2_ spraying. Photosynthesis was allowed to stabilise under the desired CO_2_ mole fraction (at 250 µmol m^−2^ s^−1^ PAR) and after 4 hours, rosettes were instantly frozen and stored at –80°C for further analyses. Rosettes sampled in darkness were collected after 4 hours at 380 ppm CO_2_ and 2 hours dark-adaptation.

### Protein Extraction and In-solution Digestion

Leaf fragments were finely ground with liquid nitrogen. Protein extraction was carried out by using the TCA/acetone method. Briefly, the powder was incubated in a precipitating solution (10% TCA, 0.07 β-mercaptoethanol in acetone) for 1h at –20°C. After centrifugation (19 000 g), the pellet was rinced three times in 0.07% β-mercaptoethanol in acetone and spin-dried. It was then suspended in a solubilization solution made of 6 M urea, 2 M thiourea, 2% CHAPS (w/v) and 30 mM Tris-HCl pH 7.8 (60 µL/mg) and cell debris were eliminated by centrifugation. Total protein content was determined using the 2-D Quant-kit (GE Healthcare). 2 mg of proteins were reduced by adding DTT (final concentration: 10 mM) and then alkylated by adding iodoacetamide (final concentration: 40 mM). The samples were diluted to 1 M urea by adding 50 mM ammonium bicarbonate. Protein digestion (sequencing grade modified trypsin, Promega) was performed at an enzyme/substrate ratio of 1∶30 (w/w) by overnight incubation at 37°C, and stopped by adding 1% formic acid (v/v).

### Stable Isotope Dimethyl Labeling

Tryptic peptides were spin-dried and re-suspended in 1 mL of 5% formic acid (v/v). Stable isotope dimethyl labeling was performed according to the on-column procedure described by [47] using formaldehyde or [^2^H_2_]formaldehyde (labeling). Each sample was loaded on a separate SepPak C18 cartridge column (3cc, Waters) and washed with 0.6% acetic acid (v/v). SepPak columns were flushed seven times with 1 mL of the respective labeling reductive reagent (50 mM sodium phosphate buffer pH 7.5, 30 mM NaBH_3_CN and 0.2% CH_2_O or C^2^H_2_O (v/v)). Samples were eluted with 500 µL of 0.6% acetic acid (v/v) and 80% acetonitrile (v/v). All labelled dimethylated peptides were homogenized to form a reference sample, before being mixed with the unlabeled dimethylated peptides in a 1∶1 abundance ratio.

### Peptide Fractionation Using Strong Cation Exchange Chromatography (SCX)

Prior to SCX, the dimethyl-labeled peptides were spin-dried and resuspended in 500 µL of solvent A (30% acetonitrile (v/v), 5% formic acid (v/v), pH 2.5). SCX was performed at 200 µL/min using Zorbax BioSCX-Series II columns (0.8-mm inner diameter×50-mm length; 3.5 µm particle size) and a Famos autosampler (LC Packings). After sample loading, the first 17 min were run isocratically at 100% solvent A, followed by an increasing pH gradient using solvent B (30% acetonitrile (v/v), 5% formic acid (v/v), 540 mM ammonium formate, pH 4.7). Twelve SCX fractions per sample were automatically collected using an on-line Probot system (LC Packings).

### Selective Enrichment of Phosphopeptides Using Immobilized Metal Ion Affinity Chromatography (IMAC)

SCX fractions were dried and resuspended in 300 µL of solvent C (250 mM acetic acid, 30% acetonitrile (v/v)). Peptides were gently mixed with 80 µL of Phos-Select iron affinity gel (Sigma-Aldrich) and incubated for 2 hours using a tube rotator, as described by [48]. The mixture was transferred into SigmaPrep spin columns (Sigma-Aldrich) and the flow-through fractions containing the non-phosphorylated peptides were collected. Iron affinity gel with bound phosphopeptides was rinsed twice with 200 µL of solvent C, then with double distilled water. The elution of bound phosphopeptides was achieved with 100 µL of solvent D (400 mM NH_4_OH, 30% acetonitrile) by centrifugation at 8200 g. Eluted phosphopeptides were dried and kept at –20°C until LC-MS/MS analysis.

### LC-MS/MS Analysis

On-line liquid chromatography was performed on a NanoLC-Ultra system (Eksigent). A 4 µL sample was loaded at 7.5 µL/min on a pre-column cartridge (stationary phase: C18 PepMap 100, particles of 5 µm; column: 100 µm i.d., 1 cm length; Dionex) and desalted with 0.1% formic acid in water. After 3 min, the precolumn cartridge was connected to the separating PepMap C18 column (stationary phase C_18_ PepMap 100, particles of 3 µm; column 75 µm i.d., 150 mm length; Dionex). Buffers were 0.1% formic acid in water (solvent E) and 0.1% formic acid in acetonitrile (solvent F). Peptide separation was achieved using a linear gradient from 5 to 30% F at 300 nL/min. Eluted peptides were analysed with a Q-Exactive mass spectrometer (Thermo Electron) using a nano-electrospray interface (non-coated capillary probe, 10 µ i.d; New Objective). Peptide ions were analysed using Xcalibur 2.1 with the following data-dependent acquisition steps: (1) full MS scan on a 300 to 1400 range of mass-to-charge ratio (m/z) with a resolution of 70000) and (2) MS/MS (normalized collision energy: 30%; resolution: 17500). Step 2 was repeated for the 8 major ions detected in step 1.

### Identification of Peptides and Phosphorylation Sites

Database searches were performed using X!Tandem CYCLONE (http://www.thegpm.org/TANDEM). Cys carboxyamidomethylation and light and heavy dimethylation of peptide N-termini and lysine residues were set as static modifications while Met oxidation and phosphorylation of tyrosine, serine or threonine residues were set as variable modifications. Precursor mass tolerance was 10 ppm and fragment mass tolerance was 0.02 Th. Identifications were performed using the TAIRrelease 8 database (http://www.uniprot.org/http://www.arabidopsis.org/). Identified proteins were filtered and grouped using the X!Tandem pipeline v3.2.0 (http://pappso.inra.fr/bioinfo/xtandempipeline/). Data filtering was achieved according to a peptide E value smaller than 0.01. The false discovery rate (FDR) was estimated to 0.92%.

### Quantification of Peptides and Phosphorylation Sites

Relative quantification of non-phosphorylated peptides and phosphopeptides was performed using the MassChroQ software [20] by extracting ion chromatograms (XICs) of all identified peptides within a 10 ppm window and by integrating the area of the XIC peak at their corresponding retention time. LC-MS/MS chromatogram alignment was performed by using common MS/MS identifications as landmarks to evaluate retention time deviations along the chromatographic profiles. Alignments were performed within groups of LC-MS/MS runs originating from similar SCX fractions. For each peptide detected in the heavy and light form in a single LC-MS/MS run, a light-to-heavy ratio was computed. To compensate for possible global deviations to 1∶1 of the light/heavy ratio (i.e. unequal mixture of heavy and light samples), normalization was performed by centering to 1 the distribution of all ratios within each LC-MS/MS run. Quantitation of protein amounts was performed by averaging centered data obtained from their different peptides. Subsequent statistical analyses (analysis of variance) were performed on log_10_-transformed normalized data.

## Supporting Information

Table S1 **Eukaryotic initiation factors and ribosomal proteins indentified by nanoLC-MS/MS.**
(DOCX)

Table S2 **Phosphopeptides identified using nanoLC-MS/MS.**
(DOCX)
