# Supplementary material for: Photosynthetic Control of Arabidopsis Leaf Cytoplasmic Translation Initiation by Protein Phosphorylation
Source: PLoS One. 2013 Jul 24;8(7):e70692. doi: 10.1371/journal.pone.0070692 (PMC3722150; doi:10.1371/journal.pone.0070692)
Supplement: Table S1 — Eukaryotic initiation factors and ribosomal proteins indentified by nanoLC-MS/MS. (DOCX) [file pone.0070692.s001.docx]

**Table S1. Eukaryotic initiation factors and ribosomal proteins indentified by nanoLC-MS/MS**

| **Family** | **AgI numbers** |  |
| --- | --- | --- |
| Sa | At1g72370, At3g04770 |  |
| S2 | At1g58380, At2g41840, At1g58983, At3g57490 |  |
| S3 | At2g31610, At3g53870, At5g35530 |  |
| S3a | At3g04840, At4g34670 |  |
| S4 | At2g17360, At5g07090, At5g58420 |  |
| S5 | At2g37270, At3g11940 |  |
| S6 | At4g31700, At5g10360 |  |
| S7 | At1g48830, At3g02560 |  |
| S8 | At5g20290, At5g59240 |  |
| S9 | At5g15200, At5g39850 |  |
| S10 | At4g25740, At5g41520, At5g52650 |  |
| S11 | At3g48930, At4g30800, At5g23740 |  |
| S12 | At1g15930, At2g32060 |  |
| S13 | At3g60770, At4g00100 |  |
| S14 | At2g36160, At3g11510, At3g52580 |  |
| S15 | At1g04270, At5g09500, At5g09510, At5g43640 |  |
| S15a | At1g07770, At2g39590, At3g46040 |  |
| S16 | At2g09990, At3g04230, At5g18380 |  |
| S17 | At2g04390, At2g05220, At3g10610, At5g04800 |  |
| S18 | At1g22780 |  |
| S19 | At3g02080, At5g15520, At5g61170 |  |
| S20 | At3g47370, At5g62300 |  |
| S23 | At3g09680, At5g02960 |  |
| S24 | At3g04920, At5g28060 |  |
| S25 | At4g34555, At2g16360, At4g39200 |  |
| S26 | At2g40510, At2g40590, At3g56340 |  |
| S27 | At2g45710, At3g61110, At5g47930 |  |
| S29 | At3g43980, At3g44010, At4g33865 |  |
| P0 | At2g40010, At3g09200, At3g11250 |  |
| P1 | At5g47700 |  |
| P2 | At2g27710, At2g27720, At3g44590 |  |
| L3 | At1g43170, At1g61580 |  |
| L4 | At3g09630, At5g02870 |  |
| L5 | At3g25520, At5g39740 |  |
| L6 | At1g18540, At1g74050, At1g74060 |  |
| L7 | At2g01250, At2g44120, At3g13580 |  |
| L7a | At2g47610, At3g62870 |  |
| L8 | At2g18020, At3g51190, At4g36130 |  |
| L9 | At1g33120, At4g10450 |  |
| L10 | At1g14320, At1g26910, At1g66580 |  |
| L10a | At1g08360, At2g27530, At5g22440 |  |
| L11 | At2g42740, At3g58700, At5g45775 |  |
| L12 | At2g37190, At3g53430, At5g60670 |  |
| L13 | At3g48960, At3g49010, At5g23900 |  |
| L13a | At3g07110, At3g24830, At4g13170, At5g48760 |  |
| L14 | At2g20450, At4g27090 |  |
| L15 | At4g16720, At4g17390 |  |
| L17 | At1g27400, At1g67430 |  |
| L18 | At3g05590, At5g27850 |  |
| L18a | At1g29965, At2g34480, At3g14600 |  |
| L19 | At1g02780, At3g16780, At4g02230 |  |
| L21 | At1g09590, At1g57660 |  |
| L22 | At5g27770, At3g05560 |  |
| L23 | At2g33370, At3g04400 |  |
| L23a | At2g39460, At3g55280 |  |
| L26 | At3g49910 |  |
| L27a | At1g23290, At1g70600 |  |
| L28 | At2g19730, At4g29410 |  |
| L30 | At1g36240, At1g77940, At3g18740 |  |
| L32 | At4g18100, At5g46430 |  |
| L34 | At1g26880, At1g69620 |  |
| L38 | At2g43460 |  |
| RACK1 | At1g18080, At1g48630, At3g18130 |  |
| eIF1 | At2g04520, At5g35680 |  |
| eIF2 | At5g05470, At5g20920, At2g40290 |  |
| eIF3 | At4g11420, At5g25780, At3g56150, At4g20980, |  |
|  | At3g57290, At2g39990, At3g11400, At2g46280 |  |
| eIF4A | At3g13920, At1g54270, At1g72730, At3g19760 |  |
| eIF4B | At1g13020 |  |
| eIF4G | At5g57870, At3g60240 |  |
| eIF5 | At1g77840, At1g36730 |  |
| eIF5A | At1g13950, At1g26630, At1g69410 |  |
