# Supplementary material for: Photosynthetic Control of Arabidopsis Leaf Cytoplasmic Translation Initiation by Protein Phosphorylation
Source: PLoS One. 2013 Jul 24;8(7):e70692. doi: 10.1371/journal.pone.0070692 (PMC3722150; doi:10.1371/journal.pone.0070692)
Supplement: Table S2 — Phosphopeptides identified using nanoLC-MS/MS. (DOCX) [file pone.0070692.s002.docx]

**Table S2.** **Phosphopeptides identified using nanoLC-MS/MS**

| **Proteins Ribosomal proteins** | **Gene locus** | **Peptides^a^** | **E-value^b^** | **Charges^c^** | **MH+ Obs^d^** |
| --- | --- | --- | --- | --- | --- |
| RPS2C | At2g41840 | **(pS)^258^PYQEHTDFLASK** | 2,1E-07 | 2 | 1666,8 |
|  |  | **FSR(pS)^258^PYQEHTDFLASK** | 8,2E-04 | 3 | 2048,9 |
|  |  | AL(s)^273^(t)^274^(s)^275^KPDPVVEDQA | 9,8E-03 | 2 | 1700,9 |
| RPS3aA | At3g04840 | VFEV(pS)^69^LADLQGDEDNAYRK | 1,7E-03 | 3 | 2313,1 |
|  |  | LMDVHGDY(pS)^236^AEDVGVK | 5,5E-13 | 3 | 1878,9 |
| RPS3aA, RPS3aB | At3g04840, At4g34670 | **EA(pS)^177^SCDLK** | 3,7E-04 | 2 | 1053,5 |
| RPS6A | At4g31700 | (s)^237^RL(s)^240^SAAAKPSVTA | 1,1E-04 | 3 | 1481,8 |
|  |  | (pS)^237^RL(pS)^240^SAAAKPSVTA | 3,3E-03 | 2 | 1569,8 |
|  |  | (pS)^237^RLSSAAAKP(pS)^247^VTA | 1,7E-05 | 2 | 1569,8 |
|  |  | L(pS)^240^SAAAKPSVTA | 8,5E-04 | 2 | 1246,7 |
|  |  | SRL(pS)^240^SAAAKPSVTA | 1,3E-04 | 2 | 1489,8 |
|  |  | SRL(pS)^240^SAAAKP(pS)^247^VTA | 1,5E-03 | 2 | 1561,7 |
|  |  | SRL(pS)^240^SAAAKPSV(pT)^249^A | 4,3E-04 | 2 | 1561,7 |
| RPS6B | At5g10360 | (pS)^237^RLSSAPAKPVAA | 2,0E-05 | 3 | 1398,8 |
|  |  | (s)^237^RL(s)^240^SAPAKPVAA | 6,9E-03 | 2 | 1478,8 |
|  |  | L(pS)^240^SAPAKPVAA | 8,8E-03 | 2 | 1147,6 |
|  |  | SRL(pS)^240^SAPAKPVAA | 1,9E-06 | 2 | 1398,8 |
| RPS6A, RPS6B | At4g31700, At5g10360 | DRR(pS)^229^ESLAK | 1,3E-03 | 3 | 1205,6 |
|  |  | **DRR(pS)^229^E(pS)^231^LAK** | 9,8E-04 | 2 | 1285,6 |
|  |  | **RSE(pS)^231^LAK** | 3,7E-03 | 2 | 934,5 |
| RPS9B | At5g15200 | DLLTLDEK(pS)^68^PR | 6,1E-06 | 3 | 1430,8 |
| RPS10A | At4g25740 | **GPPR(pS)^116^DGDRPR** | 6,3E-04 | 3 | 1321,6 |
| RPS14A | At2g36160 | **VDVVTLGP(pS)^19^VR** | 2,1E-06 | 2 | 1253,7 |
| RPS14B | At3g11510 | VETVTLGP(pS)^19^VR | 1,2E-04 | 2 | 1269,7 |
| RPS17 | At1g79850 | (t)^115^K(s)^117^FVALPVIAR | 1,4E-04 | 3 | 1437,8 |
| RPS27A, RPS27B, RPS27C | At2g45710, At3g61110, At5g47930 | LVQ(pS)^29^PNSFFMDVK | 3,0E-09 | 2 | 1655,8 |
| RPP0B | At3g09200 | VEEKEE(pS)^305^DEEDYGGDFGLFDEE | 1,1E-05 | 3 | 2711,1 |
| RPP0C | At3g11250 | KEE(pS)^308^DEEDYEGGFGLFDEE | 6,7E-09 | 3 | 2367,9 |
| RPP1A, RPP1C | At1g01100, At5g47700 | **TVGELAC(pS)^10^YAVMILEDEGIAITADK** | 3,9E-11 | 3 | 2829,4 |
| RPP1A, RPP1B, RPP1C | At1g01100, At4g00810, At5g47700 | KKDEPAEE(pS)^102^DGDLGFGLFD | 8,5E-12 | 3 | 2245,1 |
|  |  | KDEPAEE(pS)^102^DGDLGFGLFD | 2,0E-08 | 2 | 2084,9 |
| RPP2A | At2g27720 | **LASVPSGGGGGVAVASAT(pS)^95^GGGGGGGAPAAESK** | 8,1E-20 | 3 | 2742,4 |
| RPP2B | At2g27710 | **LASVPSGGGGGVAVASAT(pS)^80^GGGGGGGASAAESK** | 3,9E-21 | 3 | 2732,3 |
| RPP2A, RPP2B, RPP2D | At2g27720, At2g27710, At3g44590 | EEKEE(pS)^120^DDDMGFSLFE | 5,6E-06 | 2 | 2050,8 |
|  |  | KEEKEE(pS)^120^DDDMGFSLFE | 3,1E-09 | 3 | 2211,0 |
| RPP3A, RPP3B | At4g25890, At5g57290 | KKEE(pS)^90^EEEEGDFGFDLFG | 6,6E-04 | 3 | 2268,0 |
|  |  | KEE(pS)^90^EEEEGDFGFDLFG | 5,1E-10 | 2 | 2107,9 |
| RPL3A | At1g43170 | **WAQHL(pS)^28^EEVRR** | 3,7E-03 | 2 | 1564,7 |
|  |  | **QYD(pS)^139^EDGKK** | 9,1E-07 | 2 | 1245,6 |
|  |  | **FQT(pS)^377^LEK** | 9,1E-03 | 2 | 988,5 |
| RPL6B, RPL6C | At1g74060, At1g74050 | **(pS)^52^KVDAPVEKPPK** | 1,1E-06 | 3 | 1486,8 |
| RPL11A, RPL11B, RPL11C, RPL11D | At2g42740, At3g58700, At4g18730, At5g45775 | VLEQLSGQ(pT)^46^PVFSK | 4,7E-05 | 2 | 1676,9 |
| RPL13D | At5g23900 | AGDS(pT)^138^PEELANATQVQGDYMPIASVK | 6,3E-08 | 3 | 2836,4 |
| 50S ribosomal protein L1 | At3g63490 | **STRSQDLTPTPSLF(pS)^32^FA(pS)^35^SRPN** | 9,6E-03 | 3 | 2588,2 |
| RACK1B | At1g48630 | **TDG(pS)^285^TGIGNK** | 1,5E-03 | 2 | 1093,5 |
| **Eukaryotic initiation factors** |  |  |  |  |  |
| eIF5 | At1g77840 | NHS(pS)^201^DEDISPK | 7,9E-04 | 2 | 1372,6 |
| eIF5A2 | At1g26630 | M(pS)^2^DDEHHFEASESGASK | 9,3E-13 | 3 | 2007,8 |
|  |  | (pS)^2^DDEHHFEASESGASK | 2,8E-14 | 3 | 1876,8 |
| eIF5A3 | At1g69410 | **M(pS)^2^DDEHHFESSDAGASK** | 1,3E-13 | 3 | 1993,8 |
|  |  | **(pS)^2^DDEHHFESSDAGASK** | 1,5E-13 | 3 | 1862,8 |
| eIF4A1 | At3g13920 | AG(pS)^4^APEGTQFDAR | 3,4E-04 | 2 | 1418,6 |
| eIF4A1, eIF4A2 | At3g13920, At1g13020 | VHACVGG(pT)^145^SVR | 4,5E-04 | 2 | 1254,6 |
| eIF4A3 | At1g72730 | AQACVGG(pT)^147^SVR | 1,6E-07 | 2 | 1217,6 |
| eIF4B1 | At3g26400 | SS(pT)^239^FGSSFGDSGQEER | 1,1E-05 | 2 | 1789,7 |
|  |  | **QKPVERPG(pS)^462^GAGR** | 7,4E-03 | 3 | 1482,8 |
|  |  | AG(pS)^480^IDETR | 6,9E-04 | 2 | 960,4 |
| eIF4B2 | At1g13020 | **(pS)^136^YGGFDDDQR** | 4,2E-03 | 2 | 1271,5 |
|  |  | KADTEVSE(pT)^283^PTAVK | 1,1E-08 | 3 | 1651,9 |
|  |  | **AVERPG(pS)^475^SASR** | 1,7E-04 | 3 | 1228,6 |
|  |  | GGSYSERPH(pS)^489^R | 2,0E-03 | 3 | 1344,6 |
|  |  | **AG(pS)^493^IDESR** | 3,7E-03 | 2 | 946,4 |
| eIF4G | At3g60240 | **T(t)^177^(s)^178^APPNMDDQK** | 3,1E-06 | 2 | 1448,7 |
|  |  | **TT(pS)^178^APPNMDDQK** | 1,5E-09 | 2 | 1448,7 |
|  |  | **LG(pS)^530^PKDR** | 8,3E-03 | 2 | 916,5 |
|  |  | STEGSSHASSEISGS(pS)^710^PQEK | 7,8E-13 | 3 | 2136,0 |
|  |  | GP(pS)^1353^MNSSGR | 1,4E-03 | 2 | 1004,4 |
|  |  | ASDL(pS)^1508^PEVSSAR | 1,6E-04 | 2 | 1326,6 |
|  |  | QVLQGPSATVN(pS)^1527^PR | 1,5E-03 | 2 | 1565,8 |
| eIF3B1, eIF3B2 | At5g27640, At5g25780 | **YLVTYHSQEP(pS)^273^NPR** | 3,2E-08 | 3 | 1802,8 |
|  |  | DGEV(pS)^684^DVEEDEYEAK | 1,0E-16 | 2 | 1857,8 |
| eIF3 subunit 7 | At4g20980 | NLSNPSARPN(pT)^74^GSK | 7,9E-05 | 3 | 1586,8 |
| eIF3 subunit 7 | At5g44320 | **ALSNPSARPH(pT)^70^GSK** | 8,3E-03 | 3 | 1566,8 |
| eIF3c1 | At3g56150 | YLQSG(pS)^40^EDDDDTDTKR | 1,0E-11 | 3 | 1988,9 |
|  |  |  |  |  |  |
| eIF2B beta | At3g07300 | **TADKS(pS)^173^LTR** |  |  |  |
| eIF2B delta | At5g38640 | VAVAGAAASAV(pS)^88^PSSFSYSSR | 5,3E-16 | 2 | 2084,0 |
|  |  | VAVAGAAASAVSPS(pS)^91^FSYSSR | 1,1E-11 | 3 | 2080,0 |
|  |  | VAVAGAAASAVSPSSF(pS)^93^YSSR | 1,2E-14 | 3 | 2084,0 |
|  |  | DFPDGSTTA(pS)^108^PGR | 1,7E-11 | 2 | 1419,6 |
|  |  | RDFPDGSTTA(pS)^108^PGR | 5,2E-04 | 2 | 1575,7 |
|  |  | **SSVPVA(s)^126^(s)^127^LPGIGMDSMAAAK** | 1,3E-12 | 3 | 2120,1 |
|  |  | SSVPVAS(pS)^127^LPGIGMDSMAAAK | 2,0E-14 | 3 | 2120,1 |
|  |  | (s)^141^(s)^142^(s)^143^VPASGLTTVSVVTMPPGLSEK | 2,0E-04 | 3 | 2475,3 |
|  |  | **VAPVSSASVA(s)^218^(t)^219^(s)^220^VK** | 3,2E-03 | 2 | 1525,8 |
|  |  | **VAPVSSASVAST(pS)^220^VK** | 5,3E-05 | 3 | 1533,8 |
| TruB | At5g14460 | LYG(pS)^132^DSEDENSSR | 1,2E-06 | 2 | 1570,6 |

**Footnotes:**

a- A peptide is considered as new when absent from refs [8] and [11] and PhosphAt 4.0 database (<http://phosphat.mpimp-golm.mpg.de/>). These peptides are in bold.

b- Calculated with X!Tandem

c- Precursor charge

d- Monoisotopic protonated peptide
